# The role of IL-1 in adipose browning and muscle wasting in CKD-associated cachexia

**DOI:** 10.1038/s41598-021-94565-y

**Published:** 2021-07-23

**Authors:** Wai W. Cheung, Ronghao Zheng, Sheng Hao, Zhen Wang, Alex Gonzalez, Ping Zhou, Hal M. Hoffman, Robert H. Mak

**Affiliations:** 1 Division of Pediatric Nephrology, Rady Children’s Hospital, University of California, San Diego, 9500 Gilman Drive, MC 0831, La Jolla, CA 92093-0831 USA; 2 Department of Pediatric Nephrology, Rheumatology, and Immunology, Maternal and Child Health Hospital of Hubei Province, Tongji Medical College, Huazhong University of Science and Technology, Wuhan, China; 3 Department of Nephrology and Rheumatology, Shanghai Children’s Hospital, Shanghai Jiao Tong University, Shanghai, China; 4 Department of Pediatrics, Shanghai General Hospital, Shanghai Jiao Tong University, Shanghai, China; 5 Sichuan Provincial Hospital for Women and Children, and Affiliated Women and Children’s Hospital of Chengdu Medical College, Sichuan, China; 6 Department of Pediatrics, University of California, San Diego, USA

**Keywords:** Chronic kidney disease, Inflammation

## Abstract

Cytokines such as IL-6, TNF-α and IL-1β trigger inflammatory cascades which may play a role in the pathogenesis of chronic kidney disease (CKD)-associated cachexia. CKD was induced by 5/6 nephrectomy in mice. We studied energy homeostasis in *Il1β*^*−/−*^/CKD, *Il6*^*−/−*^*/*CKD and *Tnf*α^*−/−*^*/*CKD mice and compared with wild type (WT)/CKD controls. Parameters of cachexia phenotype were completely normalized in *Il1β*^*−/−*^/CKD mice but were only partially rescued in *Il6*^*−/−*^*/*CKD and *Tnf*α^*−/−*^*/*CKD mice. We tested the effects of anakinra, an IL-1 receptor antagonist, on CKD-associated cachexia. WT/CKD mice were treated with anakinra (2.5 mg/kg/day, IP) or saline for 6 weeks and compared with WT/Sham controls. Anakinra normalized food intake and weight gain, fat and lean mass content, metabolic rate and muscle function, and also attenuated molecular perturbations of energy homeostasis in adipose tissue and muscle in WT/CKD mice. Anakinra decreased serum and muscle expression of IL-6, TNF-α and IL-1β in WT/CKD mice. Anakinra attenuated browning of white adipose tissue in WT/CKD mice. Moreover, anakinra normalized gastrocnemius weight and fiber size as well as attenuated muscle fat infiltration in WT/CKD mice. This was accompanied by correcting the increased muscle wasting signaling pathways while promoting the decreased myogenesis process in gastrocnemius of WT/CKD mice. We performed qPCR analysis for the top 20 differentially expressed muscle genes previously identified via RNAseq analysis in WT/CKD mice versus controls. Importantly, 17 differentially expressed muscle genes were attenuated in anakinra treated WT/CKD mice. In conclusion, IL-1 receptor antagonism may represent a novel targeted treatment for adipose tissue browning and muscle wasting in CKD.

## Introduction

Cachexia, characterized by muscle wasting and frailty, has been related to increased mortality rate in patients with CKD (chronic kidney disease), cancer and chronic obstructive pulmonary disease^1,2^. A common feature of these pathological conditions is increased circulating inflammatory cytokines such as IL-1, IL-6 and TNF-α^3–5^. Systemic inflammation is associated with exaggerated skeletal muscle wasting in CKD patients as well as animal models of CKD, suggesting anti-cytokine therapy may serve as a potential strategy to treat CKD-associated cachexia^3^. Recent evidence in preclinical models suggests that blockade of IL-1 signaling may be a logical therapeutic target for chronic disease-associated muscle wasting. IL-1β activates NF-κβ signaling and induces expression of IL-6 and catabolic muscle atrophy atrogin-1 in C2C12 myocytes^6,7^. Intracerebroventricular injection of IL-1β induces cachexia and muscle wasting in mice^8^. Anakinra is a recombinant form of the natural IL-1 receptor antagonist and blocks both IL-1α and IL-1β signaling through the IL-1 receptor^9^. Anakinra was FDA-approved for the treatment of rheumatoid arthritis as well as some rare autoinflammatory disorders and is also a safe and an effective therapeutic option in a variety of diseases including diseases involving muscle. Duchene muscular dystrophy (DMD) is an X-linked muscle disease characterized by muscle inflammation that is associated with increased circulating serum levels of IL-1β. Subcutaneous administration of anakinra normalized muscle function in a mouse model of DMD^10^. Similarly, serum IL-1β is elevated in hemodialysis patients. A 4-week treatment with anakinra was shown to be safe in these patients while significantly reducing markers of systemic inflammation such as CRP and IL-6, but the effect on nutrition and muscle wasting was not established^11^. In this study, we investigated the role of inflammatory cytokines in CKD cachexia, and evaluated the efficacy of anakinra treatment in a mouse model of CKD-associated cachexia.

## Results

### Genetic deletion of *Il1β* provides a better rescue of cachexia in CKD mice compared to* Il6* and *Tnfα*

**Figure 1:**
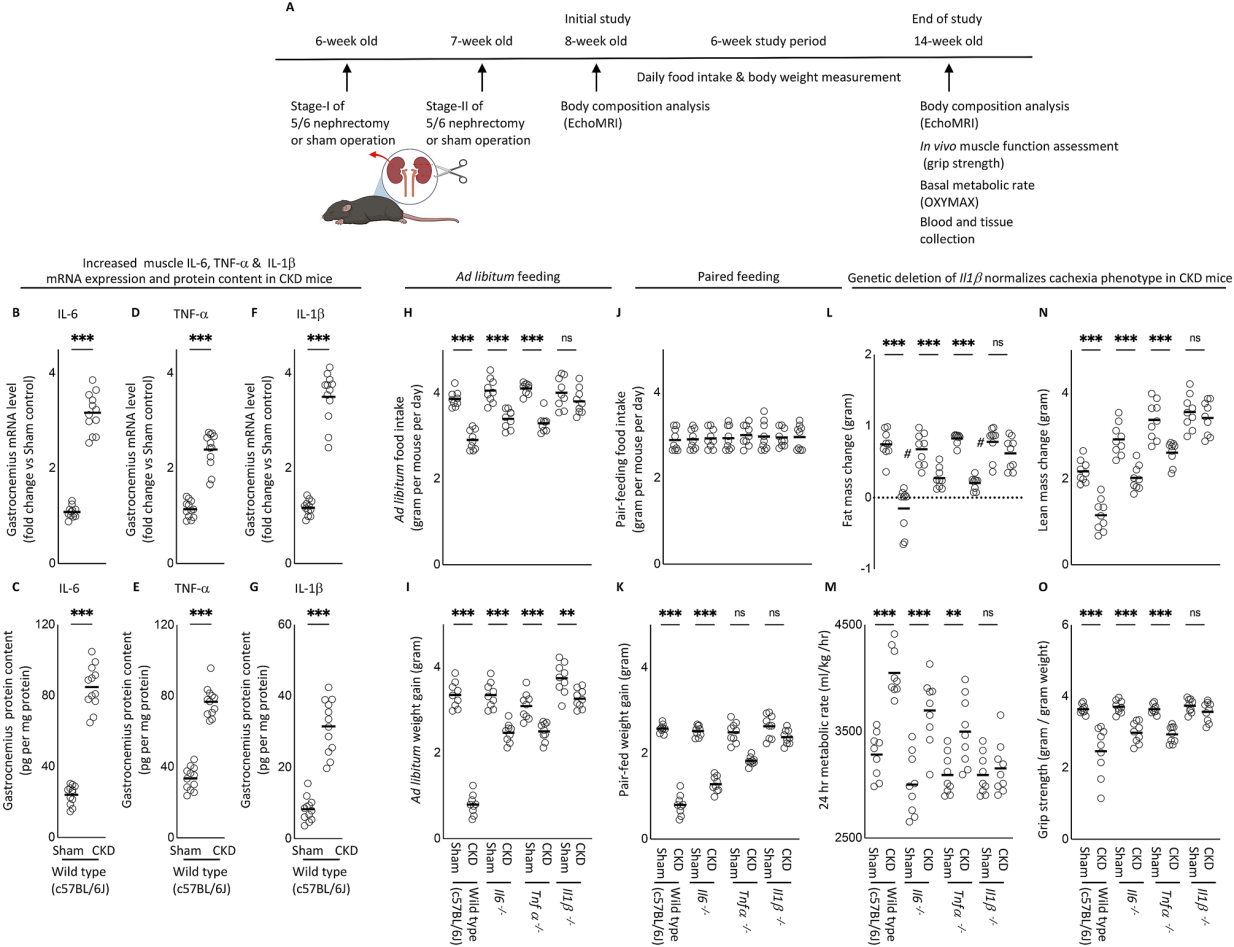
Increased muscle mRNA and protein content of IL-6, TNF-α and IL-1β in CKD mice and genetic depletion of *Il1β* provides a better rescue of cachexia in CKD mice compared to *Il6* and *Tnfα* deficiency. Schematic experimental design is shown (**A**). (**A**) Was created with BioRender.com. Results of three experiments were shown. For the first experiment, CKD was induced by 5/6 nephrectomy in WT mice and sham operation was performed in WT control mice. Gene expression and protein content of pro-inflammatory cytokine IL-6, TNF-α and IL-1β in gastrocnemius muscle in WT/CKD and WT/Sham mice was performed. Data are expressed as mean ± SEM and results of WT/CKD mice were compared to WT/Sham mice (**B**–**G**). For the second experiment, four groups of mice were included: WT/CKD and WT/Sham, *Il6*^*−/−*^*/*CKD and *Il6*^*−/−*^*/*Sham, *Tnfα*^*−/−*^*/*CKD and *Tnfα*^*−/−*^*/*Sham, *Ilβ*^*−/−*^/CKD and *Ilβ*^*−/−*^/Sham. All mice were fed ad libitum and food intake as well as weight gain in mice were recorded (**H**,**I**). For the third experiment, we investigated the metabolic benefits of genetic depletion of *Il6*, *Tnfα* and *Il1β* in CKD mice beyond the nutritional stimulation by employed a pair-feeding strategy. Four groups of mice are WT/CKD and WT/Sham, *Il6*^*−/−*^*/*CKD and *Il6*^*−/−*^*/*Sham, *Tnfα*^*−/−*^*/*CKD and *Tnfα*^*−/−*^*/*Sham, *Ilβ*^*−/−*^/CKD and *Ilβ*^*−/−*^/Sham. WT/CKD mice were fed ad libitum whereas other groups of mice were pair-fed to that of WT/CKD mice (**J**). Weight gain, fat and lean content, 24-h oxygen consumption and in vivo muscle function (grip strength) was measured in mice (**K**–**O**). For the second and third experiments, data are expressed as mean ± SEM and results of WT/CKD, *Il6*^*−/−*^*/*CKD, *Tnfα*^*−/−*^*/*CKD and *Il1β*^*−/−*^/CKD mice were compared to WT/Sham, *Il6*^*−/−*^*/*Sham, *Tnfα*^*−/−*^*/*Sham and *Il1β*^*−/−*^/Sham, respectively.

In order to determine the most important pro-inflammatory cytokines in CKD, we performed 5/6 nephrectomy in wild type (WT) as well as in *Il6*^*−/−*^, *Tnfα*^*−/−*^ and *Il1β*^*−/−*^ mice (on the same c57BL/6 J genetic background) and performed sham operation in respective control mice. All mice were 8 weeks of age, were studied for 6 weeks and sacrificed at 14-weeks of age. A schematic study design is shown in Fig. 1A. We first measured gastrocnemius muscle mRNA and protein content of IL-6, TNF-α and IL-1β in WT/CKD and compared to WT/sham mice. CKD in WT mice was evidenced by higher concentration of BUN and serum creatinine (Supplemental Table 1S). Muscle mRNA and protein content of IL-6, TNF-α and IL-1β were significantly elevated in WT/CKD compared to WT/Sham mice (Fig. 1B–G). To determine the relative functional significance of these inflammatory cytokines in CKD, we tested whether *Il6*, *Tnfα* or *Il1β* deficiency would affect the cachexia phenotype in CKD mice. WT/CKD, *Il6*^*−/−*^*/*CKD, *Tnfα*^*−/−*^*/*CKD and *Il1β*^*−/−*^/CKD mice were all uremic similar to WT/CKD mice (Supplemental Table 2S). Mice were fed ad libitum for 6 weeks and average daily food intake as well as final weight gain of the mice were recorded. We showed that food intake and weight gain were completely normalized in *Il1β*^*−/−*^/CKD mice but were only partially rescued in *Il6*^*−/−*^*/*CKD and *Tnfα*^*−/−*^*/*CKD mice (Fig. 1H,I). To investigate the potential metabolic effects of genetic deficiency of *Il6*, *Tnfα* or *Il1β* beyond its nutritional effects, we employed a pair-feeding strategy. WT/CKD mice were fed ad libitum and then all other groups of mice were fed the same amount of rodent diet based on the recorded WT/CKD food intake (Fig. 1J). Despite receiving the same amount of total calorie intake as other groups of mice, cardinal features of cachexia phenotype such as decreased weight gain, decreased fat mass content and hypermetabolism (manifested as elevated oxygen consumption), decreased lean mass content and reduced muscle function (grip strength) were evident in WT/CKD mice (Fig. 1K–O). Importantly, the cachexia phenotype was completely normalized in *Il1β*^*−/−*^/CKD mice relative to *Il1β*^*−/−*^/Sham mice whereas *Il6*^*−/−*^*/*CKD and *Tnfα*^*−/−*^*/*CKD mice still exhibited some degree of cachexia.

### Anakinra attenuates cachexia in CKD mice

**Figure 2:**
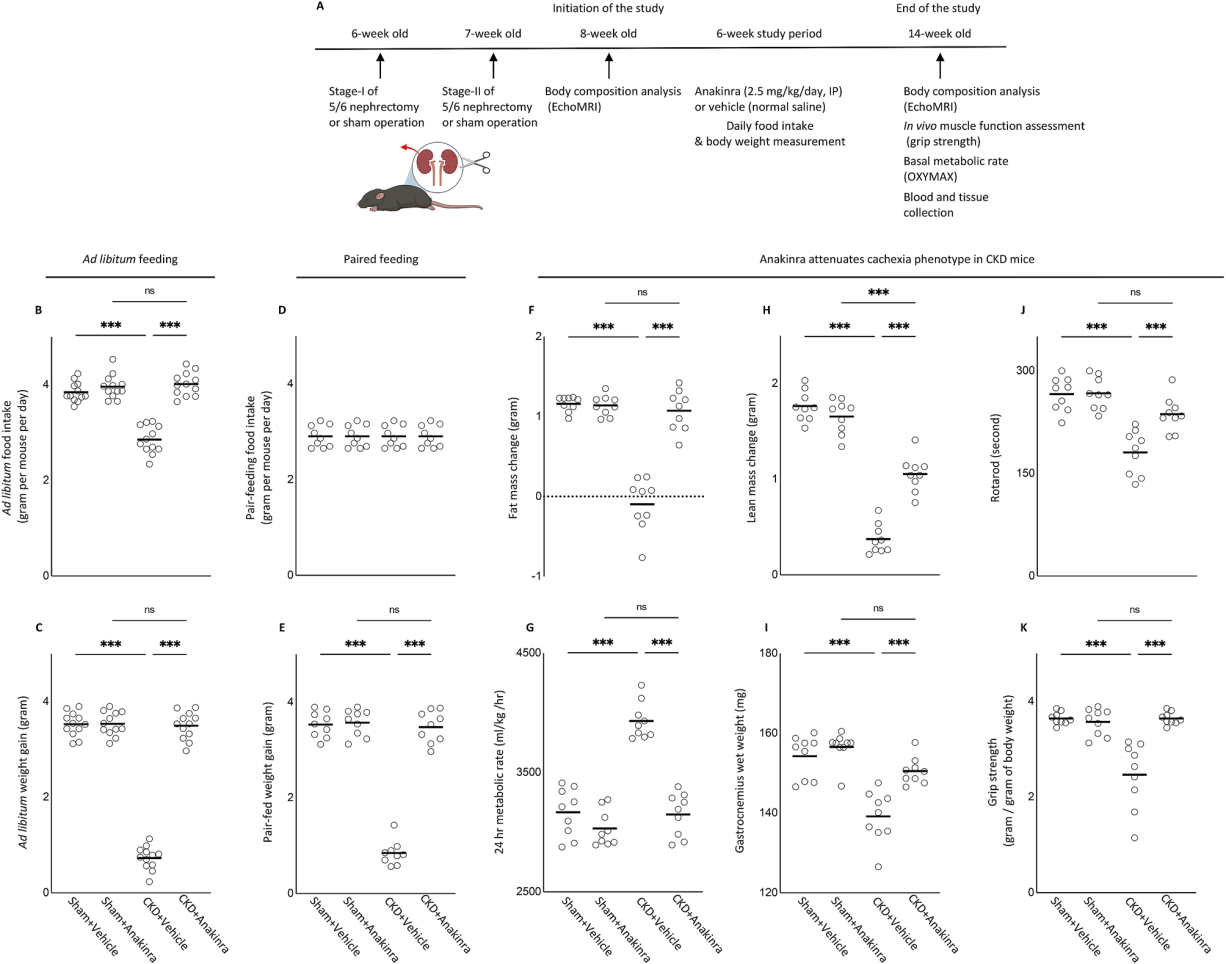
Anakinra attenuates cachexia in CKD mice. WT/CKD and WT/Sham mice were treated with anakinra (2.5 mg/kg/day, IP, daily) or normal saline as a vehicle control for 6 weeks. Schematic experimental design is shown (**A**). Figure 2A was created with BioRender.com. Results Mice were fed ad libitum and food intake and weight gain in mice were recorded (**B**,**C**). To assess the beneficial effects of anakinra beyond its nutritional effects, we employed a pair-feeding strategy. WT/CKD mice treated with vehicle were given an ad libitum amount of food whereas other groups of mice were given an equivalent amount of food (**D**). Weight change, fat and lean mass content, 24-h oxygen consumption and in vivo muscle function (grip strength) was measured in mice (**E**–**K**). Data are expressed as mean ± SEM. Results of WT/CKD + Vehicle were compared to WT/Sham + Vehicle and WT/CKD + Anakinra were compared to WT/Sham + Anakinra, respectively. In addition, results of WT/CKD + Anakinra were also compared to WT/CKD + Vehicle. ^*^ p < 0.05, ^**^ p < 0.01, ^***^ p < 0.001.

Based on the rescue of CKD phenotype in *Il1β*^*−/−*^ mice, we tested the efficacy of anakinra as a therapy for cachexia using a CKD mouse model. Experimental design is shown in Fig. 2A. Eight-week-old WT/CKD and WT/Sham mice were treated with anakinra or vehicle for 6 weeks and were sacrificed at 14-weeks of age. All mice were fed ad libitum. Food intake and weight gain were normalized in anakinra treated WT/CKD mice (Fig. 2B,C). We also investigated the beneficial effects of anakinra in WT/CKD mice beyond appetite stimulation and its consequent body weight gain through the utilization of a pair-feeding approach. Daily ad libitum food intake for WT/CKD mice treated with vehicle was recorded. Following that, anakinra treated WT/CKD mice were food restricted such that the mice ate an equivalent amount of food as vehicle treated WT/CKD mice (Fig. 2D). Serum and blood chemistry of mice were measured (Supplemental Table 3S). Anakinra normalized or attenuated weight gain, metabolic rate, fat and lean mass content, gastrocnemius weight, as well as in vivo muscle function (rotarod and grip strength) in WT/CKD mice (Fig. 2E–K).

### Anakinra attenuates serum concentration and muscle expression of IL-6, TNF-α and IL-1β in CKD mice

**Figure 3:**
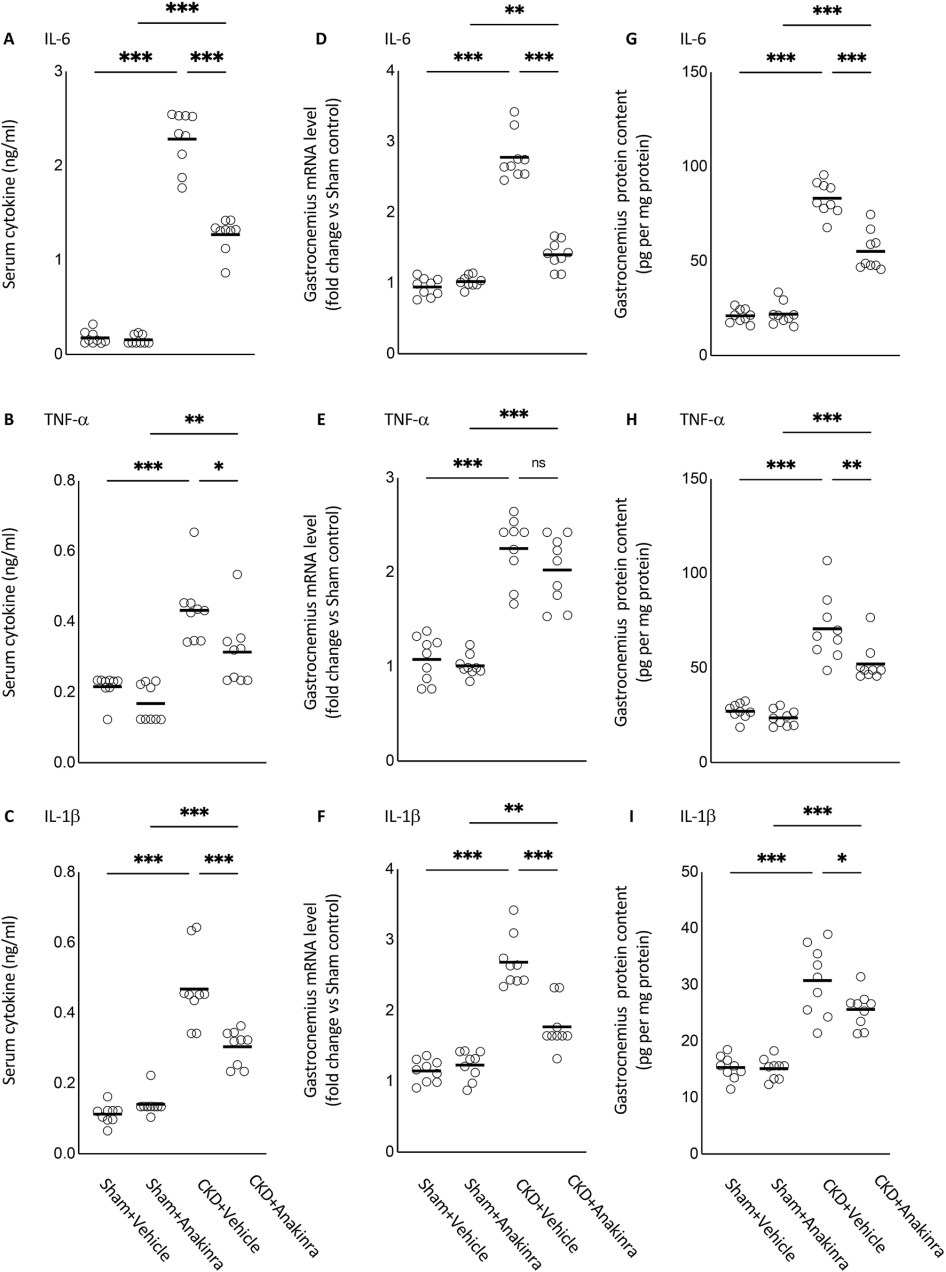
Anakinra attenuates serum and muscle expression of IL-6, TNF-α and IL-1β in CKD mice. At the end of 6 weeks of anakinra or vehicle treatment, WT/CKD and WT/Sham mice were sacrificed and serum cytokine concentration (**A**–**C**) as well as gastrocnemius muscle mRNA levels (**D**–**F**) and protein content (**G**–**I**) of IL-6, TNF-α and IL-1β were measured. Data are expressed as mean ± SEM. Results of WT/CKD + Vehicle were compared to WT/Sham + Vehicle and WT/CKD + Anakinra were compared to WT/Sham + Anakinra, respectively. In addition, results of WT/CKD + Anakinra were also compared to WT/CKD + Vehicle. ^*^ p < 0.05, ^**^ p < 0.01, ^***^ p < 0.001.

Serum concentration of IL-6, TNF-α and IL-1β as well as gastrocnemius muscle mRNA and protein content of IL-6, TNF-α and IL-1β were significantly increased in WT/CKD compared to WT/Sham mice (Fig. 3). We showed that anakinra decreased serum concentration of IL-6, TNF-α and IL-1β as well as muscle mRNA and protein content of IL-6 and IL-1β in WT/CKD mice. In addition, anakinra treatment significantly decreased muscle protein content of TNF-α in WT/CKD mice.

### Anakinra normalizes energy homeostasis in skeletal and adipose tissue in CKD mice

**Figure 4:**
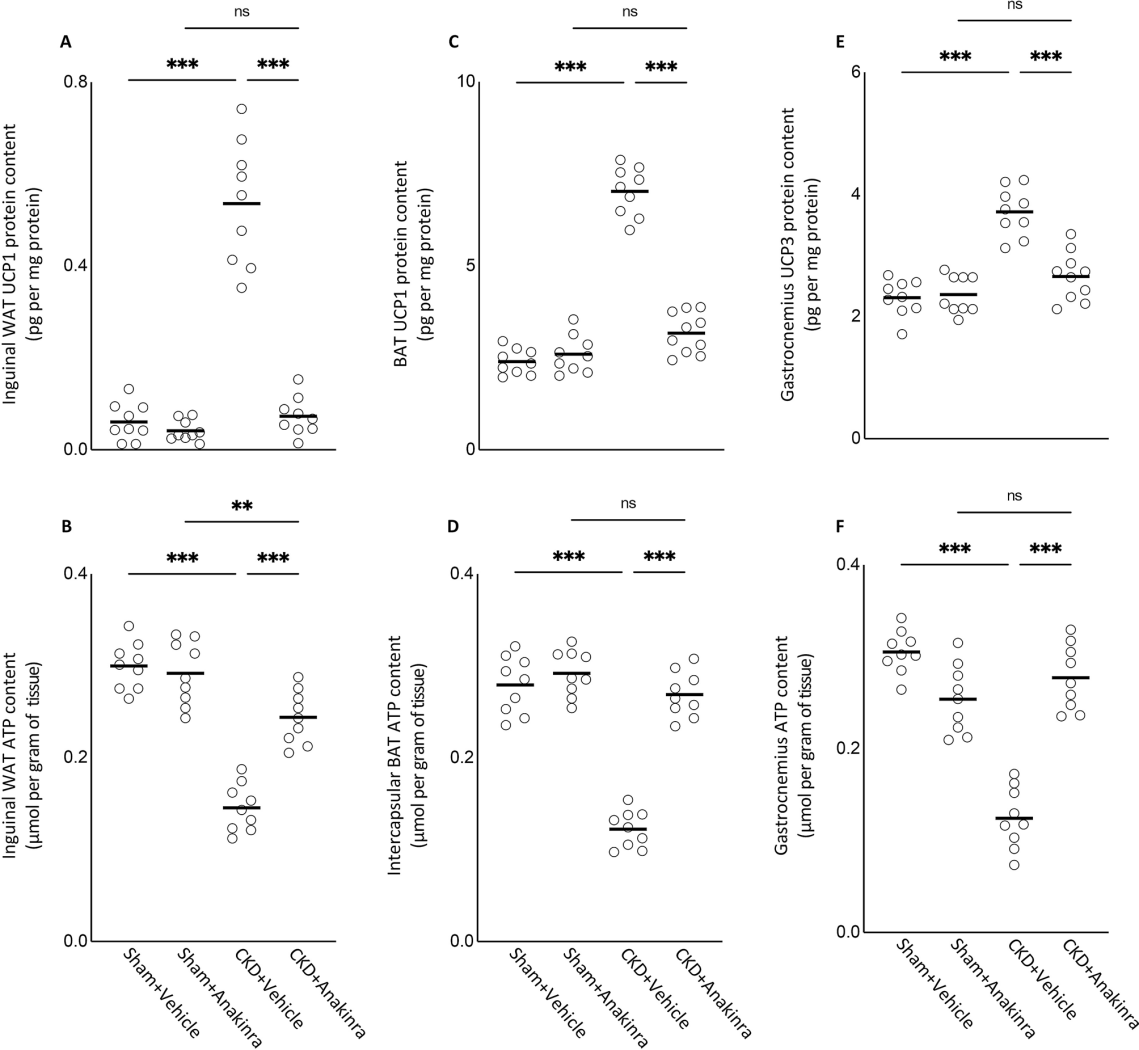
Anakinra ameliorates energy homeostasis in skeletal and adipose tissue in CKD mice. UCP content (**A**,**C**,**E**) and ATP protein content (**B**,**D**,**F**) in adipose tissue (inguinal white adipose tissue and brown adipose tissue) and gastrocnemius muscle were measured. Final results were expressed in arbitrary units, with one unit being the mean level in WT/Sham + Vehicle mice. Results of WT/CKD + Vehicle were compared to WT/Sham + Vehicle and WT/CKD + Anakinra were compared to WT/Sham + Anakinra, respectively. In addition, results of WT/CKD + Anakinra were also compared to WT/CKD + Vehicle. ^*^ p < 0.05, ^**^ p < 0.01, ^***^ p < 0.001.

We studied energy homeostasis by measuring content of uncoupling proteins (UCPs) and ATP in mouse adipose tissue and gastrocnemius muscle. UCP1 content of inguinal white adipose tissue (WAT) and intercapsular brown adipose tissue (BAT) as well as UCP3 content in gastrocnemius was significantly increased in WT/CKD mice versus control (Fig. 4A,C,E). In contrast to UCP content in WT/CKD mice, decreased ATP content in WAT and BAT as well as gastrocnemius was observed in WT/CKD mice versus control (Fig. 4B,D,F). Anakinra normalized or attenuated contents of UCPs and ATP in WAT and BAT of WT/CKD mice.

### Anakinra ameliorates browning of white adipose tissue in CKD mice

**Figure 5:**
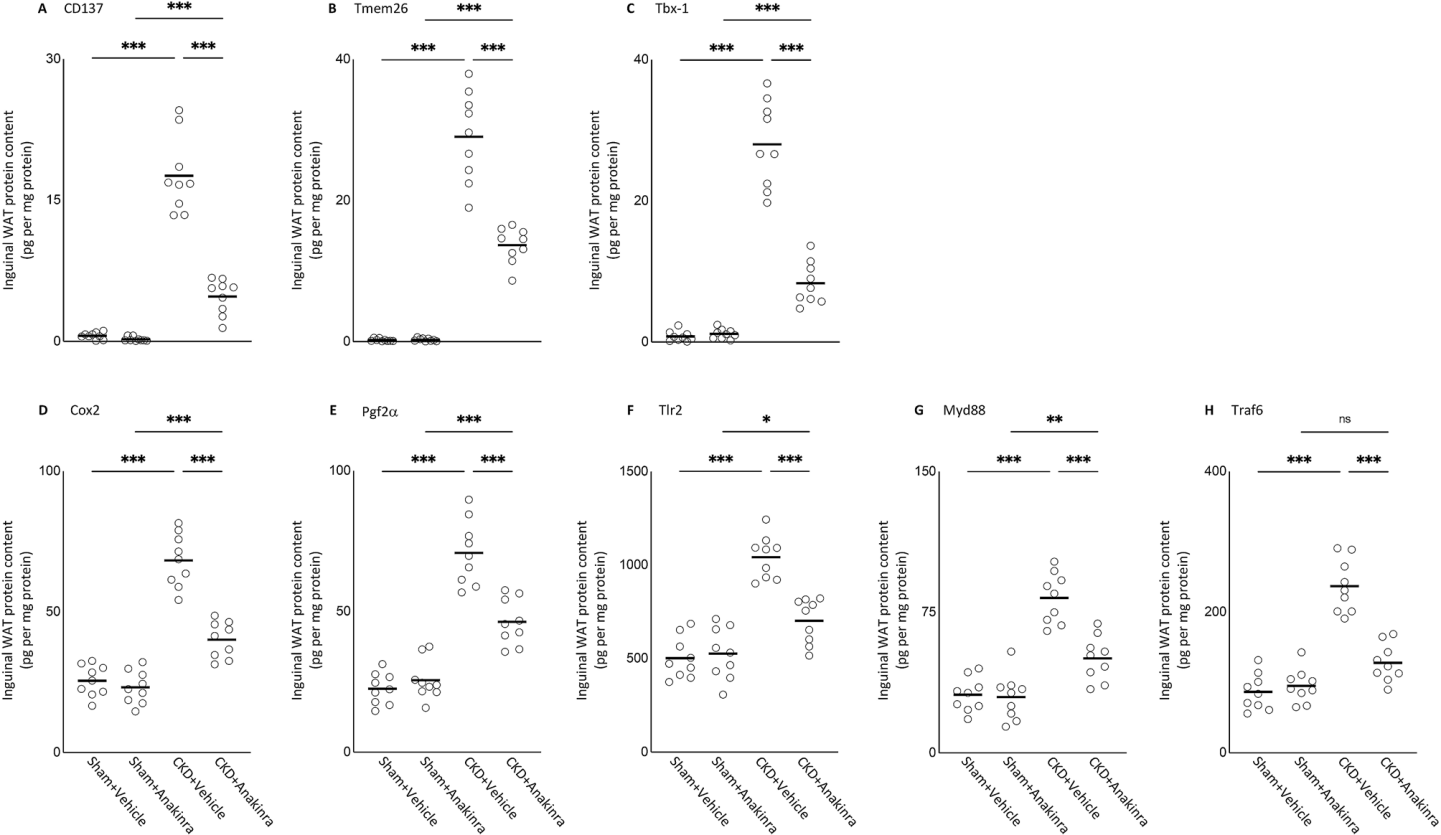
Anakinra attenuates adipose tissue browning in CKD mice. Protein content of beige adipocyte markers (CD137, Tmem26 and Tbx-1) in inguinal white adipose tissue was measured (**A**–**C**). In addition, protein content of Cox2 signaling pathway (Cox2 and Pgf2α) and toll like receptor pathway (Tlr2, MyD88 and Traf6) in inguinal white adipose tissue was measured (**D**–**H**). Results of WT/CKD + Vehicle were compared to WT/Sham + Vehicle and WT/CKD + Anakinra were compared to WT/Sham + Anakinra, respectively. In addition, results of WT/CKD + Anakinra were also compared to WT/CKD + Vehicle. ^*^ p < 0.05, ^**^ p < 0.01, ^***^ p < 0.001.

Browning of WAT regulates systemic energy homeostasis. Beige adipocytes are distinct cells in mouse and human which exhibit a unique gene expression profile, in comparison to white adipocytes or brown adipocytes. Beige adipocytes reside in WAT deposits and display intermediate characteristics of both WAT and BAT. Our results suggest the presence of beige adipocytes in WAT of WT/CKD mice. Expression of beige adipocyte markers, including UCP1, CD137, Tmem26, and Tbx1*,* were detected in WAT of WT/CKD mice (Fig. 4A, Supplemental Fig. 1 & Fig. 5A–C). We characterized the expression of several key transcriptional regulators of brown adipogenesis in WAT of WT/CKD mice. Increased expression of Cox2, Pgf2α, Tlr2, Myd88 and Traf6 was observed in WAT of WT/CKD mice relative to control mice (Supplemental Fig. 1 & Fig. 5D–H). Anakinra attenuated expression of beige adipocyte markers and transcriptional regulators of adipose tissue browning in inguinal WAT of WT/CKD mice relative to vehicle treated controls.

### Anakinra attenuates signaling pathways implicated in muscle wasting in CKD mice

**Figure 6:**
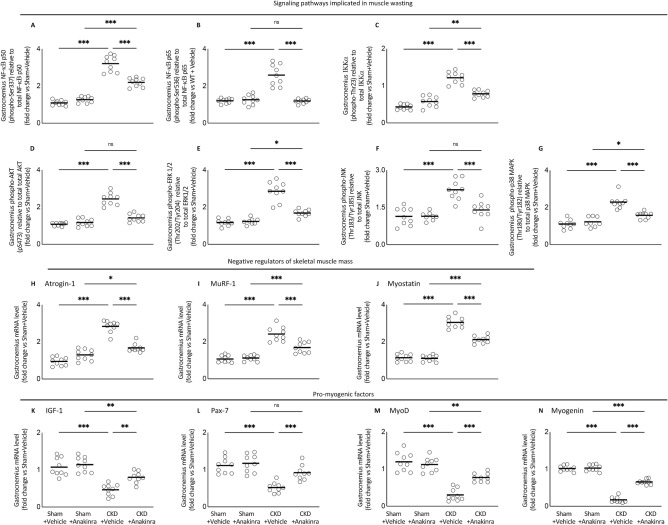
Anakinra attenuates signaling pathways implicated in muscle wasting in CKD mice. Gastrocnemius muscle relative phosphorylated NF-κB p50 (Ser337) / total p50 ratio (**A**), NF-κB p65 (Ser536) / total p65 ratio (**B**) and Iκκ*α* (Thr23) / total Iκκ*α* ratio (**C**) as well as muscle relative phospho-Akt (pS473) / total Akt ratio (**D**), ERK 1/2 (Thr202/Tyr204) / total ERK 1/2 ratio (**E**), JNK (Thr183/Tyr185) / total JNK ratio (**F**), p38 MAPK (Thr180/Tyr182) / total p38 MAPK ratio (**G**) in mice. In addition, gastrocnemius muscle expression of interested genes in mice was measured by qPCR. Transcriptional expression of negative regulators of skeletal muscle mass (Atrogin-1, MuRF-1 and Myostatin) (**H**–**J**) and pro-myogenic factors (IGF-1, Pax-7, MyoD and Myogenin) (**K**–**N**) were expressed in arbitrary units, with one unit being the mean level in WT/Sham + Vehicle mice. Results of WT/CKD + Vehicle were compared to WT/Sham + Vehicle and WT/CKD + Anakinra were compared to WT/Sham + Anakinra, respectively. In addition, results of WT/CKD + Anakinra were also compared to WT/CKD + Vehicle. ^*^ p < 0.05, ^**^ p < 0.01, ^***^ p < 0.001.

We characterized the expression of several important signaling pathways that regulate skeletal muscle metabolism in CKD mice. Increased expression of NF-κB (Fig. 6A–C) and AKI signaling pathway (Fig. 6D–G) was evident in gastrocnemius of WT/CKD mice. This is accompanied by the increased expression of negative regulators of skeletal muscle mass (Atrogin-1, Murf-1 and Myostatin) (Fig. 6H–J) but decreased expression of pro-myogenic factors (IGF-1, Pax-7, MyoD and Myogenin) in gastrocnemius of WT/CKD mice (Fig. 6K–N). Anakinra attenuated or normalized expression of these regulators in gastrocnemius of WT/CKD mice.

### Anakinra improves muscle fiber size and attenuates muscle fat infiltration in CKD mice

**Figure 7:**
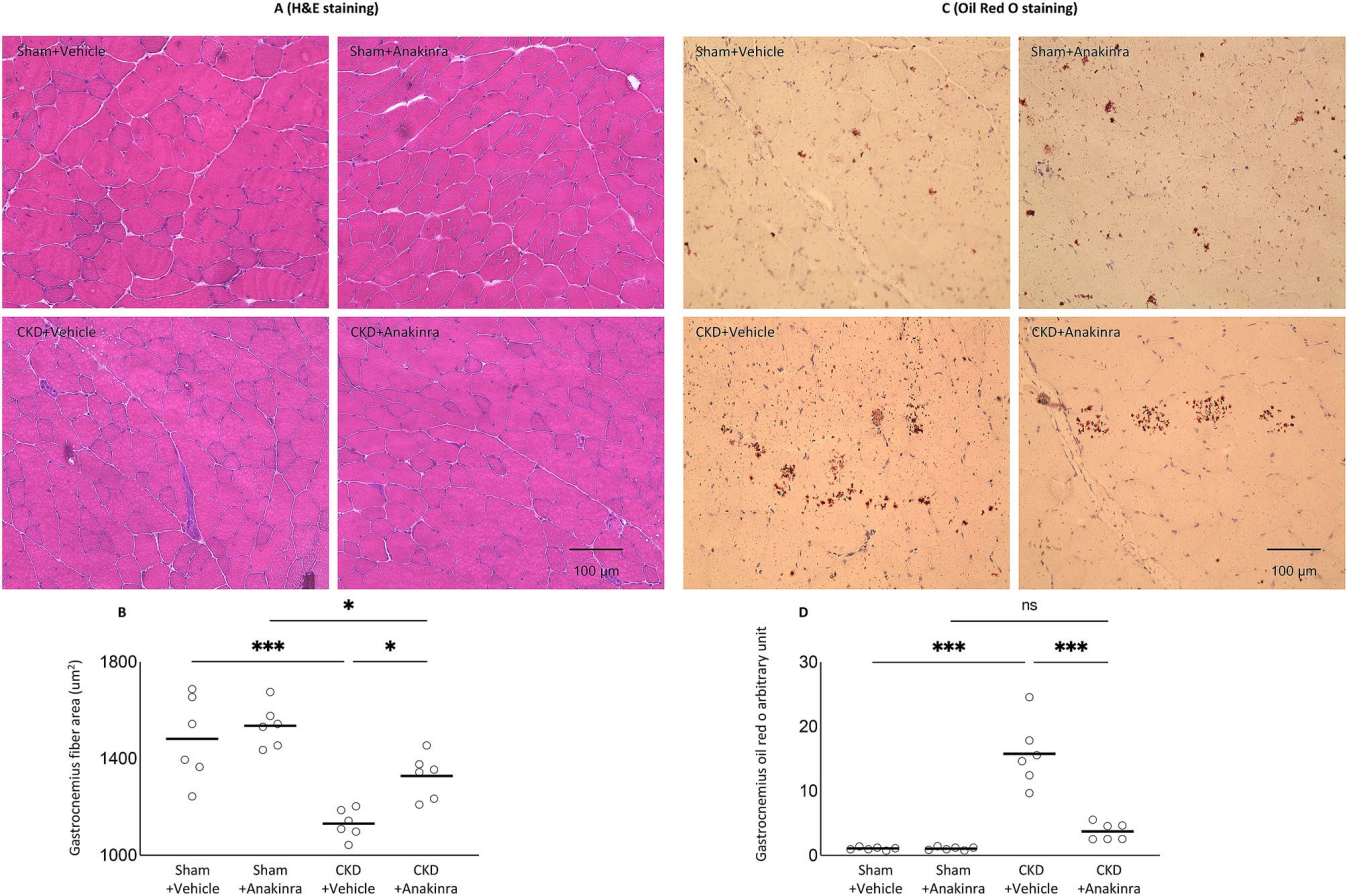
Anakinra normalizes muscle fiber size and attenuates muscle fat infiltration in CKD mice. Representative photomicrographs of gastrocnemius with H&E staining (**A**). Average gastrocnemius cross-sectional area was measured (**B**). Visualization of quantification of fatty infiltration by Oil Red O analysis in gastrocnemius muscle (**C**,**D**). Final results were expressed in arbitrary units, with one unit being the mean staining intensity in vehicle-treated WT/Sham mice. Difference among various groups of mice were analyzed as in Fig. 2.

Immediately after sacrificing of the mice, we isolated the gastrocnemius muscle and measured the cross-sectional areas of myofibers and quantified fatty infiltration. We found that anakinra attenuated cross-sectional areas of myofibers and fatty infiltration in gastrocnemius of WT/CKD mice (Fig. 7).

### Molecular mechanism by RNAseq analysis

**Figure 8:**
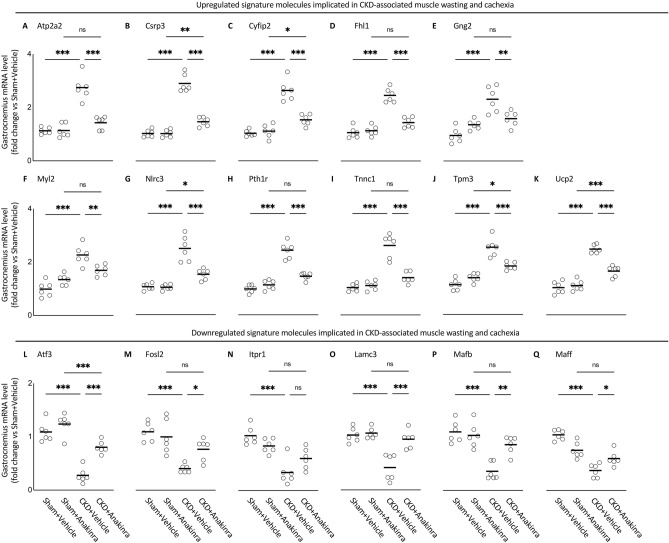
Anakinra attenuates expression of the top 17 differentiated expression gastrocnemius muscle genes in CKD mice. Gastrocnemius muscle expression of interested genes in mice was measured by qPCR. Transcriptional expression of upregulated signature molecules implicated in CKD-associated muscle wasting and cachexia (Atp2a2, Csrp3, Cyfip2, Fhl1, Gng2, Myl2, Nlrc3, Pth1r, Tncc1, Tpm3 and Ucp2) (**A**–**K**) and downregulated signature molecules implicated in CKD-associated muscle wasting and cachexia (Atf3, Fosl2, Itpr1, Lamc3, Mafb and Maff) (**L**–**Q**) were expressed in arbitrary units, with one unit being the mean level in WT/Sham + Vehicle mice. Results of WT/CKD + Vehicle were compared to WT/Sham + Vehicle and WT/CKD + Anakinra were compared to WT/Sham + Anakinra, respectively. In addition, results of WT/CKD + Anakinra were also compared to WT/CKD + Vehicle. ^*^ p < 0.05, ^**^ p < 0.01, ^***^ p < 0.001.

Previously, we performed transcriptomic profiling of gastrocnemius muscle mRNA in 12-month-old WT/CKD mice versus age-appropriate WT/Sham mice by RNAseq analysis and identified 20 differentially expressed genes in muscle^12^. In this study, 8-week-old WT/CKD and WT/Sham mice were treated with anakinra or vehicle for 6 weeks and all experimental animals were sacrificed at 14-weeks of age. We studied the effects of anakinra on skeletal muscle and energy homeostasis in 14-week-old CKD mice, focusing on these top 20 differentially expressed muscle genes identified in our previous investigation. Anakinra attenuated or normalized expression of 17 out of 20 muscle genes in WT/CKD mice (Fig. 8 and Table 1) while expression of three muscle genes remained significantly elevated in WT/CKD mice (Supplemental Fig. 2).

**Table 1:** Anakinra normalizes or attenuates expression of important muscle genes that have been implicated in muscle wasting in CKD mice.

Upregulated differential expressed genes	Functional significance & references
Atp2a2	Associated with myogenic differentiation in slow-twitch muscle fiber^57^
Csrp3	Associated with skeletal muscle dystrophy^47^
Cyfip2	Associated with muscle wasting^48^
Fhl1	Activates myostatin signaling and promotes atrophy in skeletal muscle^49^
Gng2	Biomarker of fatty infiltration in muscle^61^
	Associated with adipocyte morphology and metabolic derangements^61^
Myl2	Associated with muscle wasting by inhibiting myoblast proliferation^51^
Nlrc3	Implicated in skeletal muscle wasting by inhibiting cell proliferation and promoting cell apoptosis^52^
Pth1r	Associated with muscle fatigue, cardiovascular pathology and hyperparathyrodism^56^
	Implicated in parathyroid-hormone-dependent skeletal muscle mass metabolism^53^
Tnnc1	Regulates straited muscle contraction^54^
	Implicated in cardiomyopathy pathogenesis and age-related skeletal muscle wasting^54^
Tpm3	Promotes slow myofiber hypotrophy and associated with generalized muscle weakness^55^
Ucp2	Regulates body metabolism and implicated in skeletal muscle wasting^62,63^
Downregulated differential expressed genes	Functional significance & references
Atf3	Impairs motor neuron survival and muscle innervation^58^
Fosl2	Implicated in FoxO-dependent gene network in muscle wasting^54^
Itpr1	Impairs muscle regeneration^59^
Lamc3	Impairs muscle regeneration and induces muscle dystrophy^60^
Mafb	Implicated with Duane retraction syndrome in patients with focal segmental glomerulosclerosis^65^
Maff	Implicated in Prdm4-induced white adipose tissue browning^64^

Previously, we studied differential expression of gastrocnemius mRNA between 12-month-old WT/CKD mice and WT/Sham mice using RNAseq analysis^12^. We focus on pathways related to energy metabolism, skeletal and muscular system development and function, nervous system development and function as well as organismal injury and abnormalities. For this study, WT/CKD and WT/Sham mice were treated with anakinra or vehicle for 6 weeks and mice were sacrificed at 14-weeks of age. We studied the effects of anakinra on skeletal muscle and energy homeostasis in CKD mice by extrapolating the top 20 differentially expressed muscle genes identified in our previous investigation. Importantly, anakinra normalized (Atf3, Atp2a2, Fhl1, Fosl2, Gng2, Itpr1, Lamc3, Mafb, Maff, Myl2, Nlrc3, Pth1r, Tnnc1, Tpm3, Ucp2) and attenuated (Csrp3), Cyfip2) muscle gene expression in WT/CKD mice relative to WT sham mice. Functional significance of each of these 17 differentially expressed muscle genes is listed.

## Discussion

Up to 40% of patients with advanced CKD exhibit the signs of cachexia. Cachexia in patients with CKD is a serious clinical consequence and is associated with greater morbidity and mortality^13,14^. Inflammation plays a major role in many disease-associated cachexia. Specifically, the inflammatory cytokines, IL-6, TNF-α and IL-1 have been implicated in CKD-associated cachexia. Our studies using specific cytokine deficient mice and the IL-1 targeted therapy anakinra suggest that IL-1 is the most important cytokine in CKD associated cachexia. We showed that anakinra normalized or attenuated food intake and weight gain, fat and lean mass content, metabolic rate and muscle function in CKD mice. Anakinra also attenuated serum and muscle expression of IL-6, TNF-α and IL-1β in CKD mice. Moreover, anakinra attenuated browning of white adipose tissue in CKD mice. Furthermore, anakinra normalized gastrocnemius weight and fiber size as well as attenuated muscle fat infiltration in CKD mice. This was accompanied by correcting the increased muscle wasting signaling pathways while promoting the decreased myogenesis process in gastrocnemius of CKD mice. Together, our results suggest that anakinra may be an effective targeted treatment approach for cachexia in patients with CKD.

Peripheral or central administration of IL-1β suppresses food intake, activates energy metabolism and reduces weight gain in experimental animals^8,15^. IL-1β signals through the appetite-regulating neuropeptides such as leptin and suppresses appetite^16^. Previously, we have demonstrated that elevated circulating concentration of leptin through the activation of melanocortin receptor 4 induces CKD-associated cachexia^17^. In this study, we showed that skeletal muscle IL-1β level was significantly increased in CKD mice, and *Il1β*^*−/−*^ mice had attenuated CKD induced cachexia (Fig. 1). In addition, anakinra improved anorexia and normalized weight gain in mice with CKD (Fig. 2B,C). Our results further highlight the beneficial effects of anakinra beyond food stimulation and accompanied weight gain. In pair-fed studies in which CKD and control mice were fed the same amount of food, cachexia was attenuated in CKD mice treated with anakinra compared to vehicle treated mice (Fig. 2E).

IL-1β is a crucial inflammatory mediator that regulates many downstream inflammatory cytokines such as TNF-α and IL-6, but also regulates its own expression in what has been described as an autoinflammatory process. This feature has been described in many inflammatory disorders^18,19^. In this current study, anakinra attenuated the serum protein concentration and muscle mRNA expression of IL-6, TNF-α and IL-1β in CKD mice (Fig. 3). Anakinra has been shown to control systemic and tissue inflammation in multiple diseases including rare inherited recurrent fever disorders as well as more common and complex autoinflammatory disorders such as gout and Still’s disease^20^. The beneficial effects of administration of anakinra in CKD mice were in agreement with human data in the context of CKD-associated cachexia. Deger *et al.* have shown that systemic inflammation, as assessed by increased serum concentration of CRP, is a strong and independent risk factor for skeletal muscle wasting in CKD patients^3^. Their results provide rationale for further studies using anti-cytokine therapies for patients with CKD. Administration of anakinra reduced inflammatory response in CKD patients as reflected by significant decreases in plasma concentration of inflammatory biomarkers CRP and IL-6^11^. A subsequent study by the same group also showed that blockade of IL-1 significantly reduced inflammatory status (decreased plasma concentration of IL-6, TNF-α and Nod-like receptor protein 3) as well as improved antioxidative property (increased plasma concentration of superoxide) in patients with stages 3–5 CKD^21^.

Loss of adipose tissue is a crucial feature of cachexia and is associated with increased lipolysis or decreased adipogenesis. Adipogenesis, the formation of adipocytes from stem cells, is important for energy homeostasis and is involved in processing triglycerol, the largest energy reserve in the body^22^. IL-1β inhibits adipogenesis as suggested by the finding that potential of adipogenic progenitor cells isolated from patients with DMD are significantly reduced when co-cultured with IL-1β-secreting macrophages^23^. We showed that anakinra normalized fat content in CKD mice (Fig. 2F). The basal metabolic rate accounts for up to 80% of the daily calorie expenditure by individual^24^. Skeletal muscle metabolism is a major determinant of resting energy expenditure^25,26^. IL-1β increases basal metabolic rate (as represented by an increase in resting oxygen consumption) in a dose-dependent manner^27^. In our study, anakinra normalized the increased 24-h metabolic rate in CKD mice (Fig. 2G).

Adipose tissue UCP1 expression is essential for adaptive adrenergic non-shivering thermogenesis and muscle UCP3 level controls body metabolism^28^. The energy generated when dissipating the proton gradient via upregulation of UCPs is not used for cellular ATP production or other biochemical processes but instead to produce heat^29,30^. Anakinra normalized or attenuated contents of UCPs and ATP in WAT and BAT of WT/CKD mice (Fig. 4). Blockade of IL-1 receptor signaling may also mitigate the metabolic dysfunction through leptin signaling.

Adipose tissue browning is associated with profound energy expenditure and weight loss in CKD-associated cachexia^31–33^. We previously demonstrated adipose tissue browning in CKD mice (as evidenced by the detection of inguinal WAT UCP1 protein and increased expression of beige adipose cell markers CD137, Tmem26 and Tbx1)^12,34^. Activation of the Cox2 signaling pathway and chronic inflammation induce adipose tissue browning. Cox2 is a downstream effector of β-adrenergic signaling and induces biogenesis of beige cells in WAT depots^35^. In this study, we showed that anakinra attenuated inguinal WAT protein and mRNA content of Cox2 and Pgf2α level in CKD mice as well as normalized key inflammatory molecules (Tlr2, MyD88 and Traf6) involved in adipose tissue browning in CKD mice (Fig. 5 and Supplemental Fig. 1). Recent data suggest that IL1β signaling mediates adipocyte browning and thermogenesis via regulation of mitochondrial oxidative responses in both cultured human and animal adipocytes^36^.

We also investigated the effects of anakinra on muscle wasting in CKD mice. Lean mass content and gastrocnemius muscle weight was significantly decreased in CKD mice (Fig. 2H,I). We showed that anakinra normalized lean mass content, gastrocnemius weight as well as muscle function in CKD mice (Fig. 2J,K). In addition, we found that anakinra attenuated cross-sectional areas of myofibers and fatty infiltration in gastrocnemius of WT/CKD mice (Fig. 7). Muscle fatty infiltration is associated with reduced muscle strength and mobility in the elderly^37^. Muscle fat infiltration may in fact be more important than muscle lean mass content when referring to mobility function^37,38^.

IL-1 stimulates the expression of catabolic genes^4,9,39^. Several important signaling pathways regulate skeletal muscle mass metabolism. Upregulation of Akt/mTOR pathway stimulates skeletal muscle hypertrophy and atrophy^40^. Inhibition of ERK signaling attenuated muscle wasting via promoting myogenesis in tumor bearing mice^41,42^. JNK signaling is activated in mouse model of pancreatic cancer cachexia and inhibition of JNK signaling improves body weight and muscle strength (grip strength) in tumor-bearing mice^43^. MAPK are a family of protein phosphorylating enzymes that regulate a diverse aspect of cellular responses including skeletal muscle regeneration and differentiation^44–46^. NFκB is one of the most important signaling pathways linked to the loss of skeletal muscle mass in various pathophysiological conditions. Activation of NFκB in skeletal muscle leads to degradation of muscle proteins, induces inflammation and fibrosis, and blocks the regeneration of myofibers^47,48^. In this study, we showed that anakinra normalized or reduced phosphorylation of muscle Akt, ERK, JNK, MAPK, NF-κB p50 and p65 content in CKD mice (Fig. 6A,B,D–G). This was accompanied by decreasing the gene expression of negative regulators of skeletal muscle mass (Atrogin-1, Murf-1 and Myostatin) while increasing the gene expression of pro-myogenic factors (IGF-1, Pax-7, MyoD and Myogenin) in CKD mice (Fig. 6H–N).

Recently, we identified a gene expression signature by RNA sequence analysis in muscle of CKD mice compared to control mice^12^. We performed qPCR analysis for the top 20 differentially expressed muscle genes in the present study. Anakinra treatment normalized (Atf3, Atp2a2, Fhl1, Fosl2, Gng2, Itp1, Lamc3, Mafb, Maff, Myl2, Nlcr3, Pth1r, Tnnc1, Tpm3, Ucp2) and attenuated (Csrp3, Cyfip2) gene expression in muscle from CKD mice (Fig. 8). Aberrant gene expression of Csrp3, Cyfip2, Fhl1, Fosl2, Myl2, Nlcr3, Pth1r, Tnnc1 and Tpm3 have been implicated in muscle atrophy and muscle weakness^49–57^. Parathyroid hormone (PTH) and its receptor may mediate the crosstalk between adipose tissue and muscle in CKD cachexia. Parathyroid hormone 1 receptor (PTH1R) functions as a receptor for PTH and PTH-related peptide (PTHrP)^58^. PTH and PTHrP, which signal through the same receptor Pth1r, induce adipose tissue and muscle wasting in murine models of cancer and CKD^31,33^. Increased expression of Pth1r has been associated with muscle fatigue, cardiovascular pathology and hyperparathyroidism as well as skeletal muscle wasting^55,58^. CKD mice in this study had elevated circulating PTH levels but anakinra did not normalize serum PTH levels in CKD mice (Supplemental Table 3S), suggesting that PTH/PTHrP pathway may not be the only mediator of crosstalk between adipose tissue and muscle in CKD-associated cachexia. However, we did find that muscle Pth1r gene expression was significantly increased in CKD mice and anakinra normalized muscle Pth1r expression in CKD mice (Fig. 8H). Increased expression of Atp2a2 and decreased expression of Atf3, Itpr1 and Lamc3 have been associated with impaired motor neuron survival and muscle innervation, reduced myogenic differentiation and regeneration^58–62^. Moreover, increased Gng2 expression is a biomarker of fatty infiltration in muscle and increased muscle Gng2 expression has been associated with aberrant adipocyte morphology and metabolic derangements in various metabolic diseases^63^. Increased Ucp2 expression stimulates body metabolism and promotes skeletal muscle wasting^64,65^. Results also suggest that decreased expression of Maff is implicated in WAT browning^66^. Interestingly, aberrant expression of Mafb has been associated with Duane retraction syndrome in patients with focal segmental glomerulosclerosis^67^. In Table 1, we list the functional significance of each of the top 17 differentially expressed muscle genes that has been normalized or attenuated in anakinra treated CKD mice relative to control mice.

**Figure 9:**
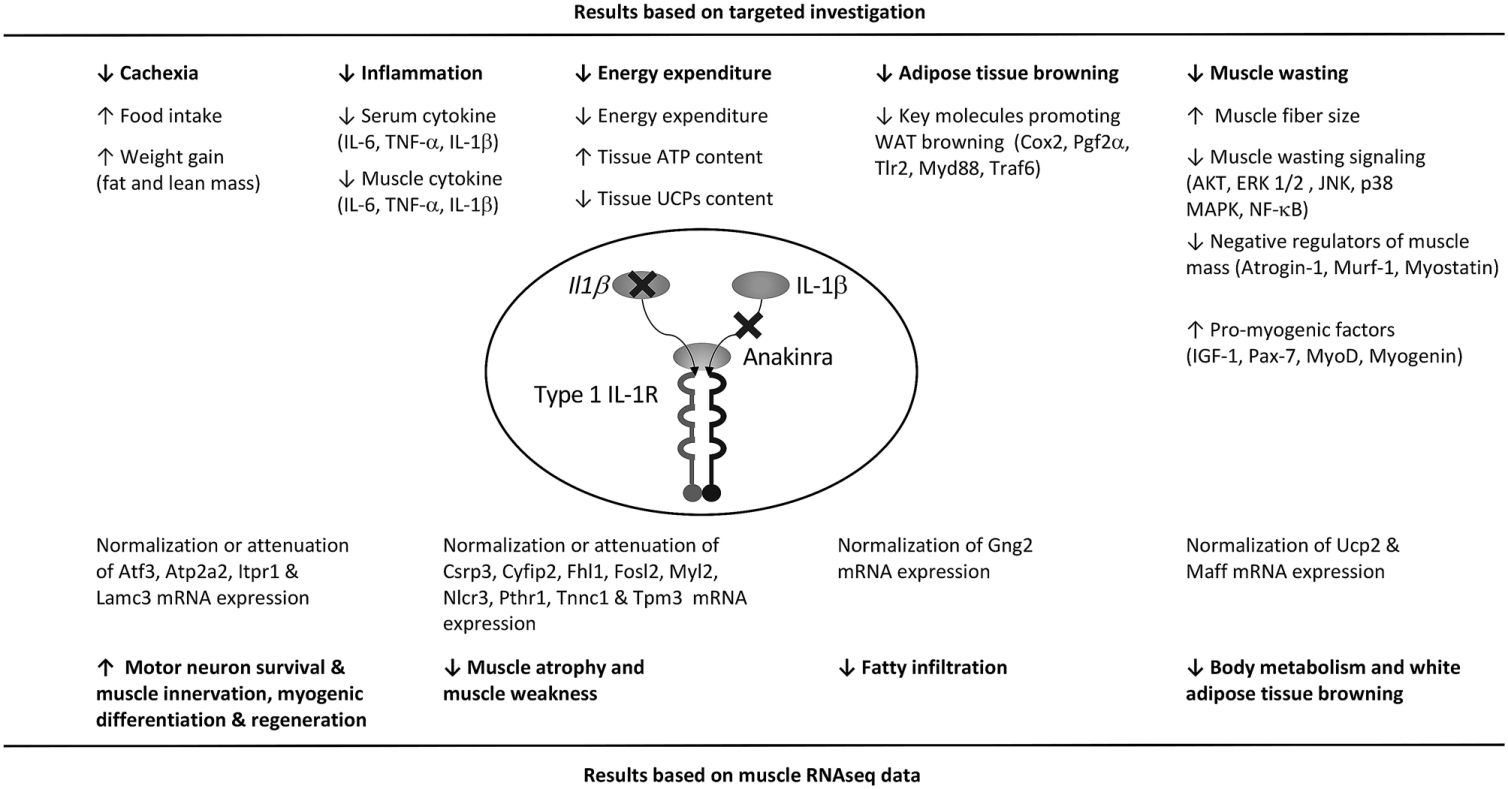
Summary of the beneficial effects of anakinra on cachexia, energy homeostasis, muscle wasting and adipose tissue browning in CKD mice.

In summary, we report that IL-1 antagonism and specific pharmacological blockade using the IL-1 receptor antagonist, anakinra, attenuates muscle wasting and adipose tissue browning in CKD mice via multiple cellular mechanisms (Fig. 9). Administration of anakinra may represent a novel targeted treatment for cachexia in CKD patients, reversing muscle wasting and adipose tissue browning, and potentially improving long term outcomes in physical functioning, quality of life and survival.

## Materials and methods

### Materials

For all reagents used in this study, please refer to Supplemental Information.

### Study design

Wild-type (WT), *Il6*^*−/−*^, *Tnfα*^*−/−*^ and *Il1β*^*−/−*^ mice were of the same c57BL/6 genetic background. Six-week-old male mice were used for the study. CKD was surgically induced by 5/6 nephrectomy in mice while sham operation was performed in respective control mice^12,68^. After the induction of CKD or sham procedure in mice, all mice were studied for 6 weeks and were sacrificed at 14-weeks of age. We have performed the following five studies. Schematic study design for study 1 to 3 was listed in Fig. 1A. Study 1—We measured gastrocnemius muscle IL-6, TNF-α and IL-1β mRNA and protein content in WT/CKD mice and pair-fed WT mice. Results were presented in Fig. 1, B to G. Study 2—We evaluated the metabolic effects of genetic deletion of *Il6*, *Tnfα* and *Il1β* in CKD mice. Specifically, we compared ad libitum food intake and weight change in WT/CKD, *Il6*^*−/−*^/CKD, *Tnfα*^*−/−*^/CKD and *Il1β*^*−/−*^/CKD mice relative to their respective controls. Results were shown in Fig. 1H,I. Study 3—We evaluated the beneficial effects of genetic deletion of *Il6*, *Tnfα* and *Il1β* in CKD mice beyond nutritional effects by employing a pair-feeding strategy. WT/CKD mice were fed ad libitum and then WT/Sham mice as well as *Il6*^*−/−*^/CKD, *Tnfα*^*−/−*^/CKD and *Il1β*^*−/−*^/CKD mice and their respective controls were fed with the same amount of rodent diet based on the recorded food intake of WT/CKD mice. Results were shown in Fig. 1J–O. Schematic study design for study 4 and 5 was listed in Fig. 2A. Study 4—We evaluated the effects of anakinra in WT/CKD mice. WT/CKD and WT/Sham mice were given anakinra (2.5 mg/kg/day, IP) or vehicle (normal saline), respectively. All mice were fed ad libitum. We compared food intake and weight change in all groups of mice. Results were shown in Fig. 2B,C. Study 5—We evaluated the metabolic effects of anakinra in WT/CKD mice beyond nutritional stimulation by employing the pair-feeding strategy. WT/CKD and WT/Sham mice were given anakinra (2.5 mg/kg/day, IP) or vehicle (normal saline), respectively. Vehicle-treated WT/CKD mice were fed ad libitum while all other group of mice were fed the same amount of rodent diet based on the recorded food intake of vehicle-treated WT/CKD mice. Results were shown in Figs. 2F–K and 3, 4, 5, 6, 7, 8. This study was performed in strict accordance with the recommendations in the Guide for the Care and Use of Laboratory Animals of the National Institutes of Health. All mice were handled according to approved institutional animal care and use committee (IACUC) protocols (S07154) of the University of California, San Diego. This study was carried out in compliance with the ARRIVE guidelines.

### Body composition, metabolic rate and in vivo muscle function

Body composition was measured by quantitative magnetic resonance analysis (EchoMRI-100™, Echo Medical System). Twenty-four-hour metabolic rate (VO_2_) was measured using Oxymax indirect calorimetry (Columbus Instrument). In vivo muscle function (grip strength and rotarod activity) in mice was assessed using a grip strength meter (Model 47,106, UGO Basile) and rotarod performance tool (model RRF/SP, Accuscan Instrument), respectively^68^.

### Serum and blood chemistry

BUN and serum concentration of bicarbonate was measured (Supplemental Table 4S). Serum creatinine were analyzed by LC–MS/MS method^69^. Serum cytokine concentration of IL-6, TNF-α and IL-1β were analyzed by Luminex assay (Biorad) according to the manufacturer’s instructions.

### Protein assay for muscle and adipose tissue

Gastrocnemius muscle, inguinal white adipose tissue (WAT) and intercapsular brown adipose tissue (BAT) were processed in a homogenizer tube (USA Scientific, catalog 1420–9600) containing ceramic beads (Omni International, catalog 19–646) using a Bead Mill Homogenizer (Omni International)^12,34^. Protein concentration of tissue homogenate was assayed using Pierce BAC Protein Assay Kit (Thermo Scientific, catalog 23227). Uncoupling protein (UCP) content as well as adenosine triphosphate (ATP) concentration in adipose tissue and muscle homogenates were assayed^12,34^. Protein contents of CD137, Tmem26, Tbx-1, Cox2, Pgf2α, Tlr2, Myd88, Traf6 in adipose tissue homogenates and protein contents of IL-6, TNF-α, IL-1β, phospho-Akt (pS473) and total Akt, phospho-ERK 1/2 (Thr202/Ty2r204) and total ERK 1/2, phospho-JNK (Thr183/Tyr185) and total JNK, phospho-p38 MAPK (Thr180/Tyr182) and total p38 MAPK, NF-κB p50 (phospho-Ser337) and total NF-κB p50, NF-κB p65 (phospho-Ser536) and total NF-κB p65, Iκκα (phosphor-Ser536) and total Iκκα in muscle homogenates were assayed^34^ (Supplemental Table 4S).

### Gastrocnemius weight, fiber size and fatty infiltration

Left gastrocnemius was excised and weighted. Fiber cross-sectional areas of left gastrocnemius were measured using ImageJ software (https://rsbweb.nih.gob/ij/). ^12^ In addition, portions of dissected right gastrocnemius muscle samples were incubated with Oil Red O (Oil Red O Solution, catalog number O1391-250 ml, Sigma Aldrich). Detailed procedures for Oil Red O staining were in accordance with published protocol^70^. We followed a recently established protocol to quantify muscle fat infiltration. Acquisition and quantification of images were analyzed using ImageJ software^71^.

### Muscle RNAseq analysis

We performed RNAseq analysis on gastrocnemius muscle mRNA in 12-month-old WT/CKD mice versus age-appropriate WT/Sham mice. Detailed procedures for mRNA extraction, purification and subsequent construction of cDNA libraries as well as analysis of gene expression was published^12^. We then performed Ingenuity Pathway Analysis enrichment tests for 20 previously identified differentially expressed muscle genes in 12-month-old WT/CKD mice versus age-appropriate WT/Sham mice, focusing on pathways related to energy metabolism, skeletal and muscle system development and function, and organismal injury and abnormalities. In this study, we performed qPCR analysis for these top 20 differentially expressed gastrocnemius muscle genes in pair-fed 14-week-old WT/CKD and WT/Sham mice treated with anakinra or vehicle, respectively.

### Quantitative real-time PCR

Total RNA from adipose and gastrocnemius muscle samples were isolated using TriZol (Life Technology) and reverse-transcribed with SuperScript III Reverse Transcriptase (Invitrogen). Quantitative real-time RT-PCR of target genes were performed using KAPA SYBR FAST qPCR kit (KAPA Biosystems). Expression levels were calculated according to the relative 2^-ΔΔCt^ method^12,68^. All primers for target genes are listed (Supplemental Table 5S).

### Statistics

Statistical analyses were performed using GraphPad Prism version 9.1.1. For comparison between two groups, data were analyzed by Student’s 2-tailed *t* test. Differences between more than two groups containing two variables were analyzed using 2-way ANOVA. *Post-hoc* analysis was performed with Tukey’s test. All data are presented as mean ± S.E.M. A *p* value less than 0.05 was considered significant.

## Supplementary Information

Supplementary Information.
