# Supplementary material for: The role of IL-1 in adipose browning and muscle wasting in CKD-associated cachexia
Source: Sci Rep. 2021 Jul 23;11:15141. doi: 10.1038/s41598-021-94565-y (PMC8302616; doi:10.1038/s41598-021-94565-y)

The role of IL-1 in adipose browning and muscle wasting in CKD-associated cachexia

Wai W Cheung[1*], Ronghao Zheng[2*], Sheng Hao[3], Zhen Wang[4], Alex Gonzalez[1], Ping Zhou[5], Hal M Hoffman[6], Robert H Mak[1]

[1] Pediatric Nephrology, Rady Children's Hospital San Diego,
University of California, San Diego
[2] Department of Pediatric Nephrology, Rheumatology, and Immunology,
Maternal and Child Health Hospital of Hubei Province, Tongji Medical College,
Huazhong University of Science and Technology, China
[3]Department of Nephrology and Rheumatology, Shanghai Children's Hospital,
Shanghai Jiaotong University, China
[4]Department of Pediatrics, Shanghai General Hospital,
Shanghai Jiaotong University, China
[5] Sichuan Provincial Hospital for Women and Children, and Affiliated Women
and Children's Hospital of Chengdu Medical College, Sichuan, China
[6] Division of Pediatric Allergy, Immunology, Rheumatology,
Rady Children's Hospital San Diego
University of California, San Diego

* These authors contributed equally to this work.

Correspondence:
Robert H Mak
Division of Pediatric Nephrology, Department of Pediatrics
University of California, San Diego
9500 Gilman Drive, MC0630, La Jolla, California 92093-0630
P: 858-822-6717
F: 858-822-6776
E-mail: romak@health.ucsd.edu

Supplemental information

(5 supplemental tables and 2 supplemental figures)

Supplemental Table 1S: Serum and blood chemistry of CKD and control mice. CKD in wild type (c57BL/6J) mice was induced by 2-stage 5/6 nephrectomy and sham operation was performed in control mice. Mice were fed *ad libitum*. Mice were sacrificed at the end of 6 weeks study and serum and blood chemistry was measured. Data are expressed as mean ± SEM. Result of serum chemistry of WT/CKD mice were compared to WT/Sham mice. # p<0.05, significantly increased in WT/CKD mice relative to WT/Sham mice.

	WT/Sham n = 12			WT/CKD n = 12		
BUN (mg/dL)	35.4	±	6.7	73.5	±	12.5 #
Creatinine (mg/dL)	0.08	±	0.02	0.24	±	0.02 #
Bicarbonate (mmol/L)	26.5	±	2.3	26.8	±	3.7
PTH (pg/mL)	123.6	±	21.5	278.6	±	23.2 #

Supplemental Table 2S: Serum and blood chemistry of *Il6*$^{-/-}$, *Tnf*$\alpha^{-/-}$, -*Il1*$\beta^{-/-}$ and wild type control mice. Genetic background of *Il6*$^{-/-}$, *Tnf*$\alpha^{-/-}$, *Il1*$\beta^{-/-}$ and wild type control mice were on c57BL/6J. CKD in *Il6*$^{-/-}$, *Tnf*$\alpha^{-/-}$, -*Il1*$\beta^{-/-}$ and WT mice were surgically induced by 5/6 nephrectomy while sham operation was performed in respective control mice. Mice were fed *ad libitum* and *e*xperiment period was 6 weeks. Data are expressed as mean ± SEM. Result of *Il6*$^{-/-}$, *Tnf*$\alpha^{-/-}$, *Il1*$\beta^{-/-}$ mice were compared to WT mice. # p<0.05, significantly increased in *Il6*$^{-/-}$*/CKD, Tnf*$\alpha^{-/-}$*/CKD* and *Il1*$\beta^{-/-}$*/CKD* mice relative to WT/sham mice.

	WT/Sham	WT/CKD	*Il6* $^{-/-}$ /Sham	*Il6* $^{-/-}$ /CKD	*Tnfa* $^{-/-}$ /Sham	*Tnfa* $^{-/-}$ /CKD	*Il1 β* $^{-/-}$ /Sham	*Il1 β* $^{-/-}$ /CKD
	n = 9	n = 9	n = 9	n = 9	n = 9	n = 9	n = 9	n = 9
BUN (mg/dL)	22.6 ± 4.6	67.8 ± 6.8 #	32.7 ± 5.8	69.1 ± 7.7 #	34.1 ± 3.8	54.7 ± 5.5 #	36.8 ± 2.5	58.9 ± 4.8 #
serum creatinine (mg/dL)	0.08 ± 0.01	0.23 ± 0.02 #	0.06 ± 0.02	0.21 ± 0.02 #	0.10 ± 0.02	0.27 ± 0.03 #	0.09 ± 0.02	0.24 ± 0.03 #
Bicarbonate (mmol/L)	27.1 ± 3.2	26.5 ± 2.6	26.8 ± 3.7	26.9 ± 2.5	28.4 ± 3.1	27.6 ± 2.4	26.9 ± 2.6	27.3 ± 3.3
PTH (pg/mL)	132.9 ± 21.3	326.7 ± 21.4 #	127.6 ± 25.7	276.8 ± 18.7 #	98.1 ± 23.7	298.6 ± 26.9 #	125.3 ± 9.6	301.6 ± 21.7 #

Supplemental Table 3S: Serum and blood chemistry of CKD and wild type control mice. WT/CKD and WT/Sham mice were given anakinra (2.5 mg/kg/day, IP) or vehicle (normal saline), respectively. Vehicle-treated WT/CKD mice were fed *ad libitum* while all other group of mice were fed the same amount of rodent diet based on the recorded food intake of vehicle-treated CKD mice. The study period was 6 weeks. Result of serum chemistry of WT/CKD+Vehicle mice were compared to WT/Sham+Vehicle mice while results of WT/CKD+Anakinra mice were compared to WT/Sham+Anakinra. Data are expressed as mean ± SEM. # p<0.05, significantly increased in WT/CKD+Vehicle and WT/CKD+Anakinra mice relative to WT/Sham+Vehicle and WT+Anakinra mice, respectively.

	WT/Sham+Vehicle<br>n = 9	WT/Sham+Anakinra<br>n = 9	WT/CKD+Vehicle<br>n = 9	WT/CKD+Anakinra<br>n = 9
BUN (mg/dL)	26.7 ± 4.7	36.1 ± 3.8	78.3 ± 9.3 #	65.8 ± 3.6 #
serum creatinine (mg/dL)	0.06 ± 0.01	0.09 ± 0.02	0.25 ± 0.02 #	0.19 ± 0.03 #
Bicarbonate (mmol/L)	27.6 ± 2.5	27.1 ± 3.1	26.7 ± 2.5	27.5 ± 4.4
PTH (pg/mL)	108.7 ± 10.6	109.6 ± 11.6	287.6 ± 7.9 #	298.4 ± 11.6 #

Supplemental Table 4S: Immunoassay information for blood and serum chemistry, muscle adenosine triphosphate content as well as muscle and adipose tissue protein analysis.

Blood & Serum chemistry	Assay information
Bicarbonate & BUN	VetScan Comprehensive Diagnostic Profile, Abaxix, 500-0038
Creatinine	LC-MS/MS method
Mouse PTH 1-84 ELISA Kit	Immutopics, 60-2305
IL-6, IL-1 beta, TNF-alpha	Bio-Rad Luminex assay

Muscle & adipose tissue	Assay information
ATP Assay Kit (Colorimetric / Fluorometric)	Abcam, ab83355
Mouse CD137 (TNFRSF9) ELISA kit	LifeSpan BioSciences, LS-F2852-1
Mouse COX2 ELISA kit	LifeSpan BioSciences, LS-F37124-1
Mouse IL-1 beta ELISA Kit	RayBiotech, ELM-IL1b-CL
Mouse IL-6 ELISA Kit	RayBiotech, ELM-IL6
Mouse TNF-alpha ELISA Kit	RayBiotech, ELM-TNFalpha
Human/mouse/rat Phospho-AKT (S473) and total AKT ELISA	Raybiotech, PEL-AKT-S473-T-1
Mouse, rat, human ERK1/2, JNK, p38 MAPK phosphorylation ELISA sampler kit	RayBiotech, CBEL-ERK-SK
Mouse IKK-alpha (phospho-Thr23) ELISA kit	LifeSpan BioSciences, LS-F1537-1
Mouse MYD88 ELISA kit	LifeSpan BioSciences, LS-F33219-1
Mouse NFKB p50 (phospho-Ser337) ELISA kit	Aviva Systems Biology, OKAG00322
Mouse NFKB p65 (phospho-Ser536) ELISA kit	RayBiotech, PEL-NFKBP65-S536-T-1
Mouse total NFKB p65 ELISA kit	RayBiotech, PEL-NFKBP65-S536-T-1
Mouse Prostagladin F2 alpha ELISA kit	MyBioSource, MBS266867
Mouse TBX1 ELISA kit	Antibodies-online, ABIN6221756
Mouse TLR2 ELISA kit	Abcam, ab224880
Mouse TNF-alpha ELISA Kit	RayBiotech, ELM-TNFalpha
Mouse TMEM26 ELISA kit	MyBiosource, MBS9319338
Mouse TRAF6 ELISA kit	LifeSpan BioSciences, LS-F53451
Mouse Ucp1 ELISA kits	Aviva Systems Biology, OKCD02970
Mouse Ucp3 ELISA kits	Aviva Systems Biology, OKEH05259

Supplemental Table 5S: PCR primer information.

Gene	Forward primer sequence	Reverse primer sequence
Atf3	GAGGATTTTGCTAACCTGACACC	TTGACGGTAACTGACTCCAGC
Atp2a2	GAGAACGCTCACACAAAGACC	CAATTCGTTGGAGCCCCAT
Atrogin-1	CAGCTTCGTGAGCGACCTC	GGCAGTCGAGAAGTCCAGTC
CD137	CGTGCAGAACTCCTGTGATAAC	GTCCACCTATGCTGGAGAAGG
Cox2	AACCCAGGGGATCGAGTGT	CGCAGCTCAGTGTTTGGGAT
Csrp3	GGGGGAGGTGCAAAATGTG	CAGGCCATGCAGTGGAAACA
Cyfip2	ATGACCACCCACGTCACTTTG	CCTGTCCTCGAAGTTCGTGTC
Fhl1	GACTGCCGCAAGCCCATAA	CCAAGGGGTGAAGGCACTT
Fosl2	CCAGCAGAAGTTCCGGGTAG	GTAGGGATGTGAGCGTGGATA
Gng2	ACCGCCAGCATAGCACAAG	AGTAGGCCATCAAGTCAGCAG
IGF-1	GTGGGGGCTCGTGTTTCTC	GATCACCGTGCAGTTTTCCA
IL-1β	GCAACTGTTCCTGAACTCAACT	ATCTTTTGGGGTCCGTCAACT
IL-6	TGGGGCTCTTCAAAAGCTCC	AGGAACTATCACCGGATCTTCAA
Itpr1	CGTTTTGAGTTTGAAGGCGTTT	CATCTTGCGCCAATTCCCG
Lamc3	CGGAGCCCTGCATCACAAA	AGCAAGGTCGTCCTCAAAGC
Mafb	TTCGACCTTCTCAAGTTCGACG	TCGAGATGGGTCTTCGGTTCA
Maff	TATGCCTTAACCTTTGGCGTC	CGATGGCAAGCGTGATGACT
Murf-1	GTGTGAGGTGCCTACTTGCTC	GCTCAGTCTTCTGTCCTTGGA
MyD88	TCATGTTCTCCATACCCTTGGT	AAACTGCGAGTGGGGTCAG
Myl2	ATCGACAAGAATGACCTAAGGGA	ATTTTTCACGTTCACTCGTCCT
MyoD	CCACTCCGGGACATAGACTTG	AAAAGCGCAGGTCTGGTGAG
Myogenin	GAGACATCCCCCTATTTCTACCA	GCTCAGTCCGCTCATAGCC
Myostatin	AGTGGATCTAAATGAGGGCAGT	GTTTCCAGGCGCAGCTTAC
Nlrc3	CAGATTGGTAACAAAGGAGCCA	CGTTCGGTTTATCTTCAGAGCA
Pax-7	TCTCCAAGATTCTGTGCCGAT	CGGGGTTCTCTCTCTTATACTCC
Pgf2α synthase	CTGGACTCATCGCAAACACAA	AGGAAGCCTTTGACTTCTGTCTA
Pth1r	CAGGCGCAATGTGACAAGC	TTTCCCGGTGCCTTCTCTTTC
Sell	TACATTGCCCAAAAGCCCTTAT	CATCGTTCCATTTCCCAGAGTC
Tbx1	CTGTGGGACGAGTTCAATCAG	TTGTCATCTACGGGCACAAAG
Tlr2	GCAAACGCTGTTCTGCTCAG	AGGCGTCTCCCTCTATTGTATT
Tmem26	TTCCTGTTGCATTCCCTGGTC	GCCGGAGAAAGCCATTTGT
Tnf	CCCTCACACTCAGATCATCTTCT	GCTACGACGTGGGCTACAG
Tnnc1	GCGGTAGAACAGTTGACAGAG	CCAGCTCCTTGGTGCTGAT
Tnni1	ATGCCGGAAGTTGAGAGGAAA	TCCGAGAGGTAACGCACCTT
Tnnt1	CCTGTGGTGCCTCCTTTGATT	TGCGGTCTTTTAGTGCAATGAG
Tpm3	ACCACCATCGAGGCGGTAA	CCCTTTCCTCCGCATCATCA
Traf6	AAAGCGAGAGATTCTTTCCCTG	ACTGGGGACAATTCACTAGAGC
Gapdh (internal control)	AGGTCGGTGTGAACGGATTTG	TGTAGACCATGTAGTTGAGGTCA

Supplemental Figure 1: Anakinra attenuates adipose tissue browning in CKD mice. Gene expression in inguinal white adipose tissue. Gene expression of beige adipocyte markers (CD137, Tmem26 and Tbx-1) in inguinal white adipose tissue was measured by qPCR. In addition, gene expression of Cox2 signaling pathway (Cox2 and Pgf2a) and toll like receptor pathway (Tlr2, MyD88 and Traf6) in inguinal white adipose tissue was measured by qPCR. Final results were expressed in arbitrary units, with one unit being the mean level in WT/Sham+Vehicle mice. Data are expressed as mean ± SEM. Results of WT/CKD+Vehicle were compared to WT/Sham+Vehicle and WT/CKD+Anakinra were compared to WT/Sham+Anakinra, respectively. In addition, results of WT/CKD+Anakinra were also compared to WT/CKD+Vehicle. * $p < 0.05$, ** $p < 0.01$.

Supplemental Figure 2: Differential expression muscle genes in CKD mice versus control mice. Gastrocnemius muscle expression of interested genes (Sell, Tnni1 and Tnnt1) in mice was measured by qPCR. Final results were expressed in arbitrary units, with one unit being the mean level in vehicle-treated WT/Sham mice. Results are analyzed and expressed as in Supplemental Figure 1.

Supplemental Figure 1

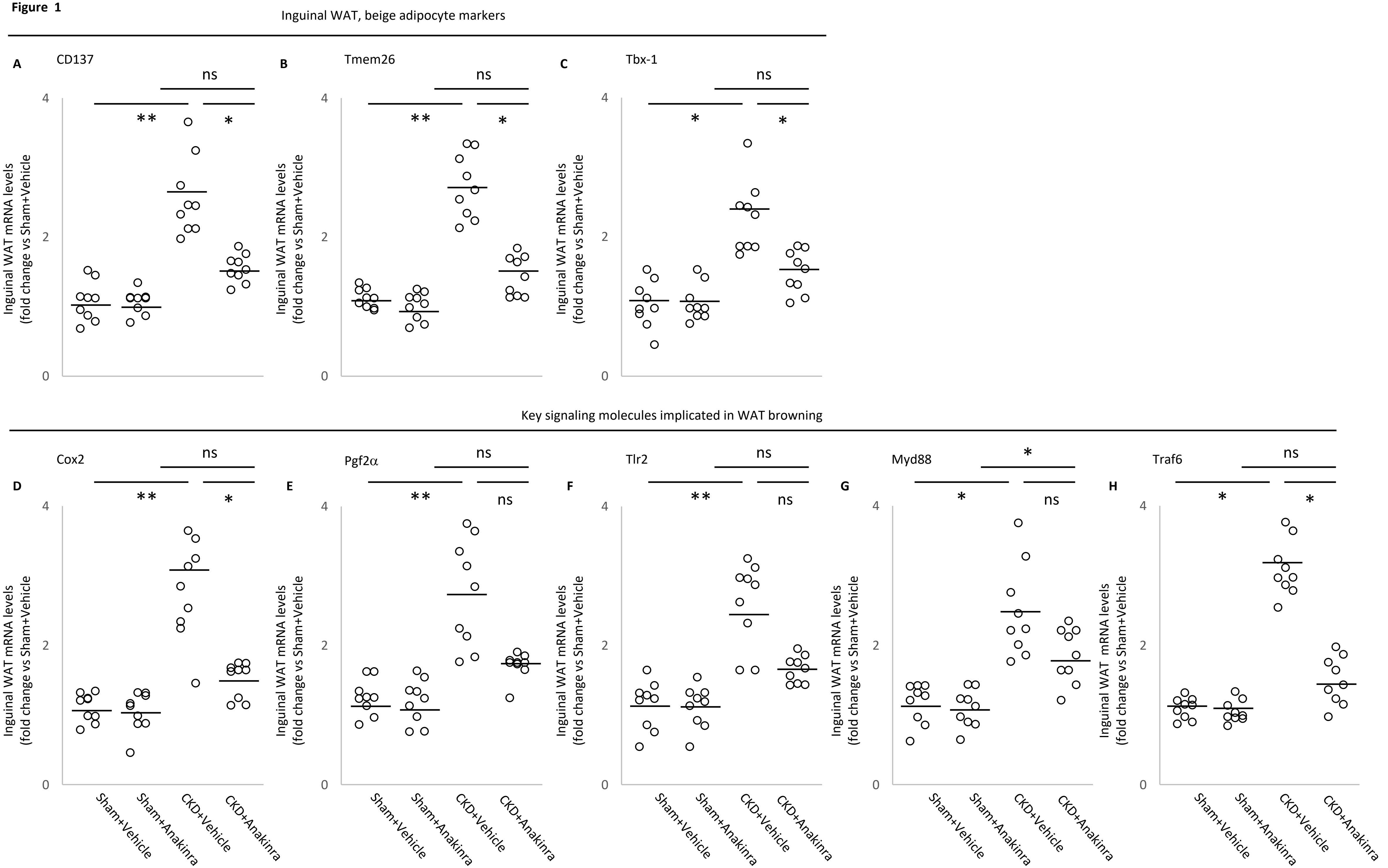

Supplemental Figure 2

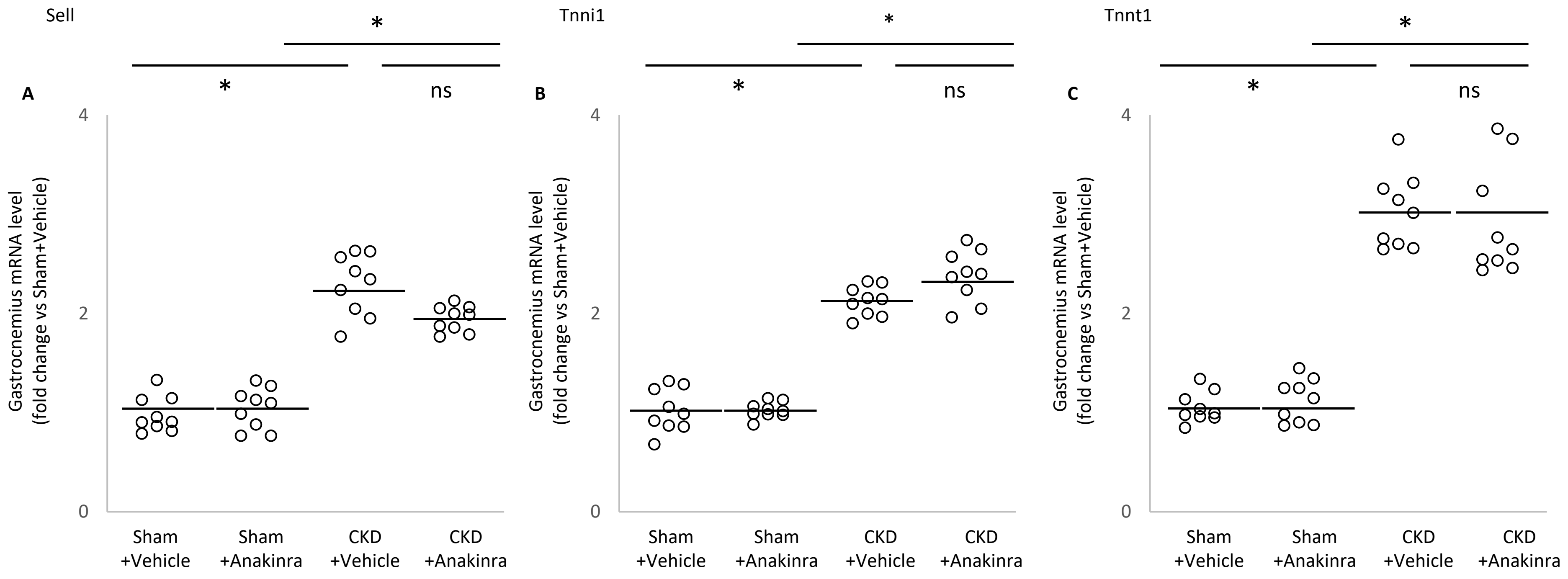

Supplement: Supplementary file 1 — Supplementary Information. [file 41598_2021_94565_MOESM1_ESM.pdf]
